# Electrochemical Salicylic Acid Sensor Based on Molecularly Imprinted Polypyrrole

**DOI:** 10.1021/acsami.5c11951

**Published:** 2025-10-02

**Authors:** Greta Zvirzdine, Sarunas Zukauskas, Alma Rucinskiene, Enayat Mohsenzadeh, Raimonda Boguzaite, Almira Ramanaviciene, Maksym Pogorielov, Vilma Ratautaite, Arunas Ramanavicius

**Affiliations:** † Department of Nanotechnology, 226274 State Research Institute Center for Physical Sciences and Technology (FTMC), Sauletekio Ave. 3, LT-10257 Vilnius, Lithuania; ‡ Department of Electrochemical Material Science, State Research Institute Center for Physical Sciences and Technology (FTMC), Sauletekio Ave. 3, LT-10257 Vilnius, Lithuania; § NanoTechnas—Center of Nanotechnology and Material Science, Institute of Chemistry, Faculty of Chemistry and Geosciences, Vilnius University (VU), Naugarduko Str. 24, LT-03225 Vilnius, Lithuania; ∥ Department of Physical Chemistry, Institute of Chemistry, Faculty of Chemistry and Geosciences, Vilnius University (VU), Naugarduko Str. 24, LT-03225 Vilnius, Lithuania; ⊥ Biomedical Research Centre, 187506 Sumy State University, Kharkivska street 116, 40007 Sumy, Ukraine; # Institute of Atomic Physics and Spectroscopy, University of Latvia, Jelgavas iela 3, LV-1004 Riga, Latvia

**Keywords:** salicylic acid (SA), molecularly imprinted polymer (MIP), density functional theory (DFT), polypyrrole (Ppy), electrochemical sensor, electrochemical overoxidation

## Abstract

This study aims to provide new insights into the development of an electrochemical salicylic acid (SA) sensor based on a molecularly imprinted polymer (MIP). Polypyrrole (Ppy) based MIP and nonimprinted polymer (NIP) layers were deposited on the platinum electrode and evaluated in a three-electrode electrochemical cell. The study used amperometry for monomer polymerization, cyclic voltammetry (CV) for the overoxidation of the polymer layer, and differential pulse voltammetry (DPV) for analyte detection. Selectivity was evaluated by comparing the electrochemical signals of SA with those of 3-hydroxybenzoic acid and melamine. Results confirm the selectivity of the electrochemical sensor. Density functional theory (DFT) calculations were performed to analyze the rebinding and recognition mechanism. The results of DFT calculations support the experimental findings. In conclusion, the polypyrrole-based MIP sensor exhibits higher selectivity and sensitivity toward salicylic acid detection compared to melamine and even to its isomer, 3-hydroxybenzoic acid (3-HBA).

## Graphical abstract

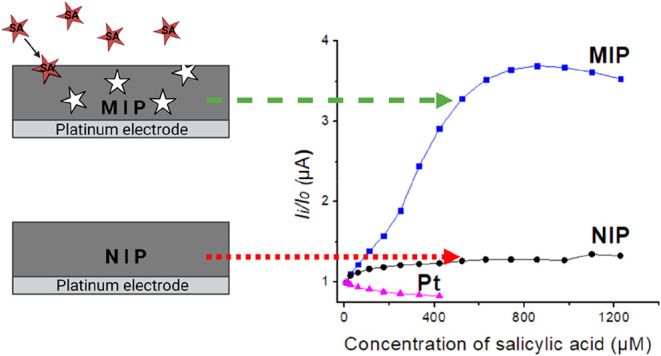

## 1 Introduction

Salicylic acid (SA) may be hazardous because an overdose of salicylic acid causes skin and respiratory tract irritation and may cause various central nervous system effects. Salicylic acid appears naturally or as a pharmaceutical pollutant in surface^1^ and ground waters^2^ or wastewater treatment plants.^3^ Additionally, water pollution has led to the detection of salicylic acid in drinking water samples.^4^ Salicylic acid is a pharmaceutical pollutant because it is the main metabolite of acetylsalicylic acid (aspirin). Aspirin plays a crucial role in various physiological processes and is a widely used drug due to its anti-inflammatory, analgesic, and antipyretic properties. It inhibits platelet aggregation and prevents blood clots, stroke, and myocardial infarction.^5^ After oral ingestion, the acetyl group in aspirin undergoes rapid hydrolysis, both enzymatically and nonenzymatically, forming salicylic acid within the body, which significantly influences the occurrence of adverse reactions.^5^ Aspirin possesses a narrow therapeutic index, creating challenges in maintaining optimal dosages, as excessive or prolonged use may lead to side effects, such as bleeding of the gastrointestinal tract.^6^ The prolonged usage of aspirin metabolite salicylic acid can lead to severe issues such as sinus and nasal inflammation, vomiting, hyperpnea, lethargy, cerebral and pulmonary edema, seizures, and multiple organ failure.^7^ Salicylate crosses the placenta, resulting in higher fetal serum concentrations, and an acute overdose in the third trimester can cause fetal death or severe toxicity.^8^ These examples demonstrate the requirement for precise monitoring.

In the food industry, salicylic acid is used because it is effective against fungi and yeast, and its antibacterial action surpasses that of benzoic acid.^9^ However, despite its benefits, salicylic acid raises some concerns regarding its safety.^10^ At the same time, some foods contain salicylic acid naturally. Salicylic acid (including salicylates) is found in many fruits, vegetables, herbs, and spices.^11^ They are part of a plant’s defense system to control biotic and abiotic stress responses.^12^ The above examples highlight the importance of SA detection in pharmaceutical monitoring and food safety.

Conventional methods for salicylic acid detection often involve complex and expensive instrumentation, such as high-performance liquid chromatography (HPLC) combined with various detection methods, including UV detection^13^ or mass spectrometry.^14^ These methods demand both highly trained personnel and costly stationary laboratory instrumentation. In this context, molecularly imprinted polymers (MIPs) have emerged as a promising alternative material.^15^ MIPs can be used to develop inexpensive, selective, and sensitive sensors. MIPs bind to and recognize specific target molecules with high affinity.^16^ They are created through a process known as molecular imprinting, where monomers surround a template molecule and then polymerize to form a three-dimensional network. Following the polymerization process, molecular imprints of the target molecule are created. After the removal of the template molecule, cavities complementary to the target molecule are formed within the polymer matrix.^17^

Computational methods emerged as a new tool that can be employed in the design of molecularly imprinted polymers (MIPs). The list of computational methods includes but is not limited to molecular mechanics, molecular dynamics, Monte Carlo simulations, quantum mechanics, and statistical simulations. Computational approaches empower the design of MIPs by providing detailed insights.^18^ This allows for scrutinising the process, optimizing the experiments, and gaining a deeper understanding of the molecular systems’ performance. Therefore, a shorter experimental process with enhanced efficiency can be achieved for complex systems, such as MIPs. Quantum mechanics simulations such as density functional theory (DFT) and classical molecular dynamics (MD) have shown remarkable potential in the design of MIPs.^19^ The description of large complex systems with high accuracy is computationally expensive. However, modeling a part of interest can provide valuable information about the host–guest system, such as noncovalent interactions and binding energies.

MIPs were applied in only several previous studies dedicated to salicylic acid determination.^20–23^ Two of these studies developed electrochemical sensors based on 4-vinylpyridine^23^ or methacrylic acid^20^ copolymers with ethylene glycol dimethacrylate. Meanwhile, studies by Zhihua et al.^21^ and Kang et al.^22^ applied polypyrrole (Ppy) and poly­(*o*-phenylenediamine), which were electropolymerised directly on the glassy carbon electrode.

This study used polypyrrole (Ppy) to develop the MIP-based electrochemical sensor for detecting salicylic acid. The novelty and significance of this study lie in addressing the need for an inexpensive and selective method to detect salicylic acid, particularly for on-site use. The sensor’s ability to discriminate salicylic acid from related compounds makes it valuable for pharmaceutical monitoring and food safety applications. In this article, we will discuss the fabrication process of the electrochemical MIP-based sensor, its characterization, and the performance evaluation in detecting salicylic acid. A density functional theory (DFT) calculation is provided to describe the mechanism of salicylic acid recognition by the MIP.

## 2 Experimental section

### 2.1 Chemicals and instrumentation

Pyrrole CAS: 109–97–7 (Alfa Aesar, Kandel, Germany), salicylic acid (2-hydroxybenzoic acid (SA)) CAS: 69–72–7, 3-hydroxybenzoic acid CAS: 99–06–9 (Thermo Fisher, Kandel, Germany), sulfuric acid CAS: 7664–93–9 (Lach-Ner, Neratovice, Czech Republic), melamine CAS: 108–78–1 (Alfa Aesar, Kandel, Germany), sodium hydroxide CAS: 1310–73–2 (StanLab, Lublin, Poland), potassium chloride CAS: 7447–40–7 (Carl Roth GmbH, Karlsruhe, Germany), redox probe (K_3_[Fe­(CN)_6_]/K_4_[Fe­(CN)_6_]): potassium hexacyanoferrate (III) CAS: 13746–66–2, (Carl Roth GmbH, Karlsruhe, Germany); and potassium hexacyanoferrate (II) (Reachim, Donetsk, Ukraine) were used in the experiments.

The μAUTOLAB TYPE III potentiostat from Metrohm (Utrecht, The Netherlands) was controlled by Nova 2.1.6 software (Utrecht, The Netherlands) and was used for electrochemical measurements. The measurements were performed in a three-electrode cell. The platinum plate (with a geometric surface area of 1.224 cm^2^) served as the working electrode, and as the counter electrode, a platinum plate was used too, and an Ag/AgCl_(3 M KCl)_ reference electrode from ItalSens (Houten, The Netherlands).

#### 2.1.1 Pretreatment of the Working Electrode

The electrodes were flame-treated to remove any organic residue and then electrochemically polished using 50 potential cycles in a 0.5 M H_2_SO_4_ electrolyte solution in the range from −1.0 to +1.0 V at the scan rate of 0.05 V/s until the signal reached equilibrium and a stable, repeating signal was obtained. The electrochemical treatment was performed in a two-electrode electrochemical cell, which contained two platinum plates: one as the working electrode and another, larger platinum plate, as the counter electrode. The pretreatment method was adapted from the previously published methods.^24^

### 2.2 Electrochemical Preparation of MIP and Nonimprinted Polymer (NIP) Layers

#### 2.2.1 Electrochemical Polymerization of NIP and MIP-Template Complex

After pretreatment, the MIP-template complex layer and NIP layers were obtained using amperometry at a constant +0.8 V vs Ag/AgCl_(3 M KCl)_ potential for 60 s in a three-electrode electrochemical cell. In this study, the MIP-template complex layer refers to the complex formed between the polymer and the target molecule before the template removal step. The MIP-template complex layer was synthesized using a solution containing 0.1 M KCl, 25 mM pyrrole, and 2.5 mM salicylic acid, and the NIP layer from a solution containing 0.1 M KCl and 25 mM pyrrole. The monomer concentration during electropolymerization significantly influences the polymerization process and is directly related to the deposition output and the quantity of template incorporated into the polymer matrix. In this experiment, the chosen monomer-to-template molar ratio was 10:1, as recommended for preparing molecularly imprinted polypyrrole-based sensors.^25^ Based on the criteria that the response difference between the MIP and NIP electrodes should be as high as possible, an optimum concentration of 25 mM was determined for the pyrrole monomer in this MIP design.^26^ Polymerization was carried out under controlled conditions with continuous stirring at 500 rpm. Stirring was employed to ensure efficient mixing of reactants and promote uniform polymerization throughout the electrode surface.

#### 2.2.2 Two-Step Template Removal Procedure

After polymerization, MIP-template complex layers were electrochemically overoxidised and then chemically treated to obtain the MIP.

#### 2.2.3 Electrochemical Overoxidation

NIP and MIP-template complex layers were overoxidised by potential cycling in a 0.1 M NaOH solution within the range of 0 to +0.6 V vs Ag/AgCl_(3 M KCl)_ at a scan rate of 50 mV/s. Both layers were cycled until they reached an equivalent conductivity, as evaluated by measuring the current density. For the MIP, this was achieved in approximately 50 potential cycles, and for the NIP, it was approximately 20 potential cycles. We chose the current density measured at the positive vertex to compare the conductivity between the MIP-template complex and NIP. Overoxidation of the polypyrrole layer increased electrochemical stability while enhancing the desorption of the analyte molecule from the MIP-template complex layer.

#### 2.2.4 Chemical Treatment for Template Removal with an Alkaline Solution

To obtain the MIP, the polypyrrole layer with embedded salicylic acid template molecules (MIP-template complex) was left in a 0.1 M NaOH solution for 15 min with stirring (200 rpm). The same procedure was performed to prepare the NIP layer. We chose to use polymer overoxidation for analyte removal based on a method described in the literature,^21^ and adapted the conditions to fit our experimental needs.

#### 2.2.5 Evaluation of MIP and NIP Layers

The layers were evaluated in a 0.1 M NaOH solution containing 5 mM of K_3_[Fe­(CN)_6_]/K_4_[Fe­(CN)_6_] as a redox couple. DPV was employed for the electrochemical analysis before and after each addition of SA concentration. After increasing the concentration of SA in the solution, we waited 5 min before the subsequent measurement. The experimental parameters for DPV: potential range from 0 to +0.45 V vs Ag/AgCl_(3 M KCl)_, pulse height of 2 mV, pulse width of 10 ms, and a scan rate of 20 mV/s. The full procedure is represented schematically in Figure 1.

**Figure 1:**
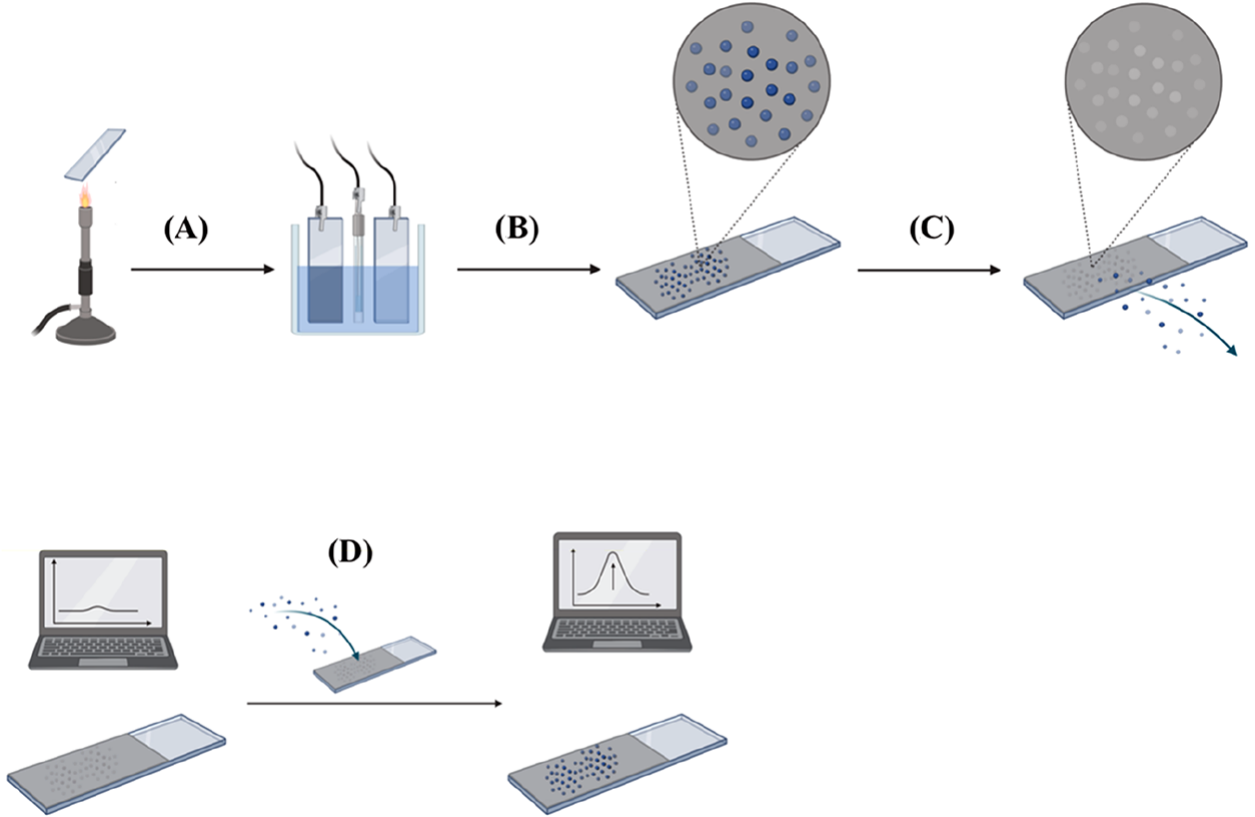
Schematic representation of experimental stages: (A) electrode pretreatment; (B) electrochemical deposition of polymer layer; (C) overoxidation of polymer layer and template removal from the MIP-template complex to obtain the MIP; (D) Electrochemical evaluation of electrochemical salicylic acid sensors.

### 2.3 DFT Calculations

The initial molecule configuration and conformation were created using the Merck molecular force field MMFF94s^27^ by the Avogadro 1.2.0 software.^28^ DFT calculations with B3LYP/def2-TZVP level of theory were performed using ORCA 5.0.4 ^29^ for geometry optimization and rebinding process in the gas phase.

The complex comprises salicylic acid as the template and five dimers of pyrrole in the oxidized form. The model represents a repeating unit of oxidized Ppy, considering the applied 10:1 monomer-to-template molar ratio in the experiments. Therefore, five repeating units are expected to imprint and interact with salicylic acid. The complementary binding sites were modeled by removing the salicylic acid template from the MIP-template complex. Next, the pyrrole dimer molecules were frozen as a firm imprinted cavity. Later, for the rebinding, salicylic acid (template), 3-hydroxybenzoic acid (isomer) and melamine were placed in the cavity. Finally, geometry optimization was performed at the same level of theory for the new complexes. This allowed the molecules to find the best position in the cavity based on their functional groups. The binding energy calculation was performed using eq 1 for the binding process of analyte molecules into the modeled binding site in the implicit (SMD) water solvent model.

1 $$E_{\mathrm{binding}}=E_{c\mathrm{omplex}}-\left(E_{\mathrm{analyte}}+E_{b\mathrm{inding site}}\right)$$

Basis set superposition error (BSSE) was treated by geometrical counterpoise (gCP) and dispersion correction by the D4 scheme.^30^ BIOVIA Discovery Studio Visualizer was used to analyze the intermolecular interactions.^31^ Noncovalent interaction (NCI) analysis was performed using Multiwfn 3.8,^32^ and the results were visualized using VMD 1.9.4.^33^

## 3 Results and Discussions

### 3.1 Electrochemical Preparation of MIP and NIP Layers

Experimental stages are presented in Figure 1.

The amperograms illustrating the formation of the NIP and the MIP-template complex layers on the electrode are shown in Figure 2A. A significant positive current offset is observed during NIP layer formation compared to the MIP-template complex layer. Further, the NIP and MIP-template complex layers were electrochemically overoxidised by potential cycling (Figure 2B) to obtain the NIP and MIP structures. Figure 2C demonstrates the current values at the upper potential vertex (+0.8 V) during electrochemical overoxidation. The decrease in the current values does not contradict the previously obtained results.^34^

**Figure 2:**
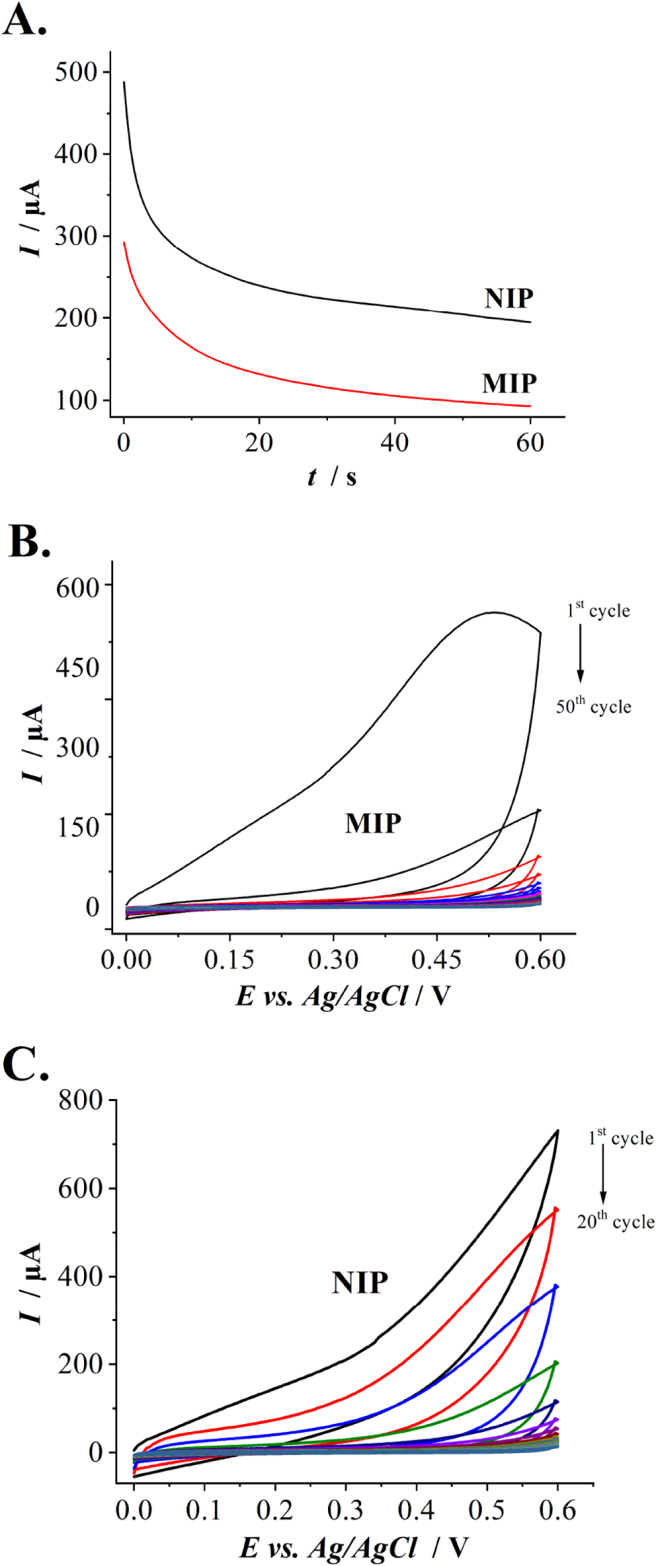
(A) Current value changes during the deposition of the NIP and the MIP-template complex layers using amperometry at the potential of +0.8 V for 60 s. (B) The cyclic voltammograms template removal from the template complex was obtained through electrochemical overoxidation by potential cycling from 0 to +0.6 V for 50 cycles in a 100 mM NaOH solution. (C) The electrochemical overoxidation of the NIP layer.

Several previous studies^35,36^ discuss a plausible explanation for the significant current difference between NIP and MIP-template complex layer deposition. Sharma et al.’s^35^ study mentions that in the case of electroactive substances used as template molecules, template molecule reaction products may be imprinted instead of genuine template molecules. The authors recommend two strategies to mitigate or eliminate this effect: (1) the usage of a dummy template (nonelectroactive compound) for imprinting, or (2) the deposition of an underlayer under the MIP-template complex layer on the electrode.^36^

Consequently, regarding the previous studies’ explanations of a similar effect, we presume that the observed negative current offset appears likely due to the addition of salicylic acid to the electrolyte solution. The electrochemical oxidation of salicylic acid occurs at approximately +0.9 V vs Ag/AgCl_(3 M KCl)_ was considered.^37^ Hence, in this study, the electrochemical polymerization of Ppy was performed using amperometry at a constant +0.8 V vs Ag/AgCl_(3 M KCl)_ potential to mitigate the inhibitory effect of salicylic acid on the MIP-template complex layer formation compared to the NIP layer formation (Figure S1). After the formation step, both polymer layers underwent a two-step template removal procedure, including electrochemical overoxidation in an alkaline solution in the first step (Figure 2B). The higher the solution’s pH, the lower the potential value needed for the polymer overoxidation.^38^ The electrochemical overoxidation application aims to succeed in template removal.^39^ Several previous studies have demonstrated that the polymer can undergo some stability issues.^40^ Although overoxidation decreases the polymer’s overall conductivity, it improves its steadiness. Simultaneously, electrochemical cycling causes mechanical deformation within the internal polymer matrix. This, combined with electrostatic forces induced by polarization, facilitates the release of the template from the MIP-template complex.

The overoxidation step is also crucial for creating a more comparable NIP to the MIP. Since NIP layer formation is more efficient than MIP-template complex layer formation, establishing an equivalent control polymer layer presents a challenge. The overoxidation of both polymer layers reduces their conductivity to similar levels, making the NIP and the MIP more comparable for subsequent measurements.

The mean MIP-template complex layer thickness of polypyrrole (γ) was estimated from the electrical charge (*q*), associated with pyrrole oxidation by application of Faraday’s Law and assuming 100% current efficiency for polypyrrole formation (eq 2)­

2 γ=qM/ρAzF

where *M* is the molar mass of the polymer, *F* is the Faraday constant, ρ is the density of the polymer, and *z* is the number of electrons involved.

The nominal density of the polypyrrole films (ρ) was taken as 1.5 g cm^–3^.^41^ Based on this value, the mean film thickness of the polypyrrole was calculated to be 72 nm. This aligns with visual observations, as only an interference pattern is visible on the surface of the platinum electrode, suggesting that the film thickness is less than the wavelength of light. However, an exact thickness could not be determined due to the surface roughness of the initial electrode. Controlling the overall thickness is critical—if the layer is too thin, it may allow nontarget molecules to diffuse toward the electrode; if it is too thick, the polymer layer could act as a barrier, reducing sensitivity.

### 3.2 Electrochemical Sensing of Salicylic Acid on MIP-Based Electrochemical Sensor

The investigation of the MIP-modified electrode revealed a linear range spanning from 8.9 to 423 μM of SA concentration. The relationship becomes nonlinear at SA concentrations starting from 523 μM. We believe that the electrochemical system begins to saturate at this concentration.

The MIP-modified electrode’s signal response to the analyte was measured using DPV with a ferro/ferricyanide redox couple. The maximum of the DPV signal was taken as the analyte response signal, from which calibration curves were subsequently generated. Since the DPV signals initially started at different currents, normalization of the signals was necessary to align all calibration curves to a common starting point. This method was selected for better observable relative changes in the signal from interaction with the analyte. The normalization process employed the following eq 2

3 $$normalised\;signal=\frac{I_{i}}{I_{0}}$$

where *I*_i_ are the current values at each salicylic acid concentration, and *I*_0_ is the current value measured in the electrolyte in the absence of salicylic acid.

As shown in Figure 3A, the produced MIP exhibits a strong concentration-dependent response to salicylic acid. The current response follows an S-shaped curve, with the highest sensitivity observed in the 200–600 μM range of salicylic acid, resulting in a significant increase in current. The saturation is reached at approximately 700 μM of salicylic acid. In contrast, the control (NIP layer) shows a slight initial response that quickly plateaus, with minimal correlation between current and concentration beyond 100 μM of salicylic acid. Meanwhile, a passivation effect was observed when using the bare platinum electrode without the NIP or MIP layers (Figure 3B).

**Figure 3:**
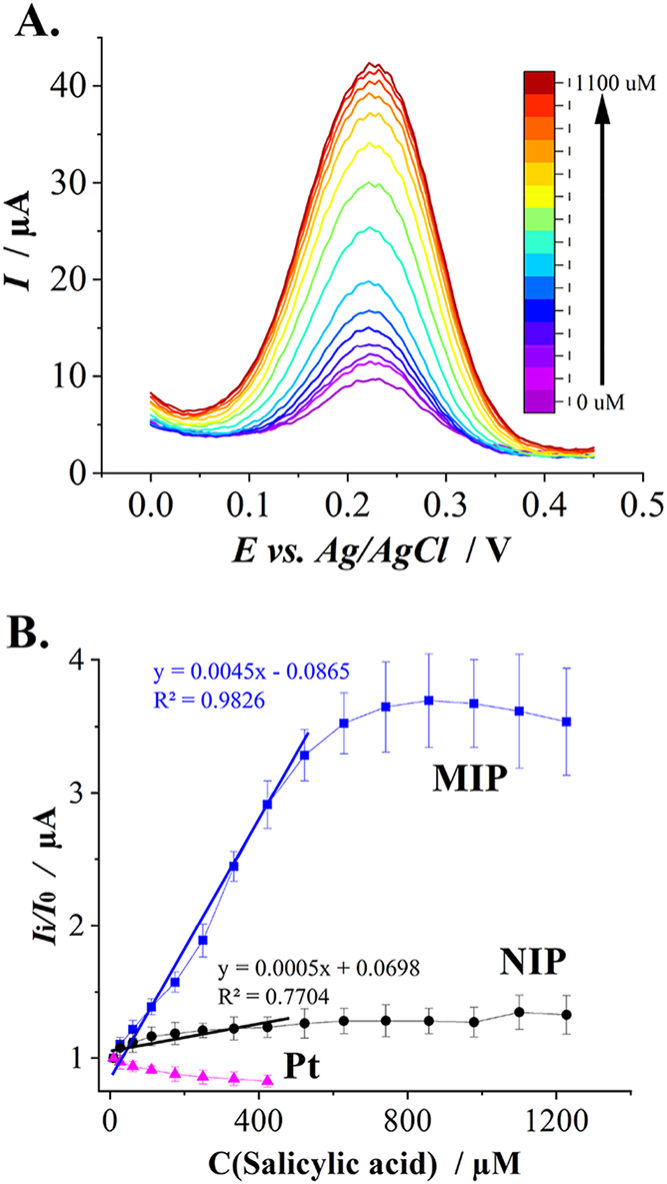
Electrochemical evaluation of MIPs’ response to salicylic acid. (A) The differential pulse voltammograms obtained using MIP in the presence of increasing salicylic acid concentration; (B) calibration curves of bare and Pt electrodes modified with MIP and NIP (MIP and NIP trendlines from 8.9 to 422.8 μM SA concentration).

One method to assess the quality of the produced MIP layers is to calculate the imprinting factor. The imprinting factor is a crucial parameter for evaluating the quality of the MIP. A certain diversity in calculation methodology is observed between different studies.^42^ We calculated the apparent imprinting factor (AIF)^43^ by comparing the ratio between the DPV signal calibration curves slopes for the MIP and NIP according to eq 4

4 $$\mathrm{AIF}=\Delta I_{s\mathrm{lope for MIP}}/\Delta I_{s\mathrm{lope for NIP}}$$

where AIF was calculated to be 9.5; values above 1 indicate successful imprinting; at a value close to 10, the result suggests a remarkably high selectivity compared to the NIP.

#### 3.2.1 Limit of Detection (LOD) and Limit of Quantification (LOQ) of the Developed Salicylic Acid Sensor

To analyze and compare differently prepared electrodes, we subtract the first DPV signal. The LOD (LOD = (3.3 × σ)/*S*, where the σ is the standard deviation and the *S* is the slope of the linear part of the calibration curve) and LOQ (LOQ = (10 × σ)/*S*) values of the MIP were calculated using the registered calibration curve. The developed sensor was characterized by LOD = 72 μM and LOQ = 217 μM, respectively, at a 95% confidence interval.

Salicylic acid (including salicylates) is naturally produced as a plant defense system against biotic and abiotic stress and is found in many fruits, vegetables, herbs, and spices.^11^ The concentration of salicylic acid may be in a broad range, and in some plants, in some food samples, with a very high content, it may exceed 1 mg/100 g. This demonstrates that an electrochemical sensor, with an LOD value of 72 μM, can find applicability in real samples.

#### 3.2.2 Selectivity Test

The sensor’s response to other molecules, such as 3-hydroxybenzoic acid (3-HBA) and melamine (ME), was electrochemically evaluated in separate solutions. The initial intention of electrochemical sensor development is a plausible screening method for milk samples to detect adulteration with salicylic acid and melamine. This is why we included melamine as an interfering molecule. However, the initial intention of the application does not limit the application of the same electrochemical sensor to other types of samples, as we mentioned in the previous question, e.g., plant samples. Each solution isolates the interactions between the sensor and a single analyte (SA, 3-HBA, or MA), ensuring that the resulting response signals can be attributed to individual analytes, improving the clarity of the data. Establishing this selectivity in simple systems is a crucial step before progressing to more complex mixtures, where factors such as matrix effects or competitive binding may impact performance. 3-HBA shares structural similarity with the target molecule SA, as they are isomers differing solely by the position of the hydroxyl (−OH) group within the molecule. We assume that the decrease in binding affinity to 3-HBA might be attributed to the slight mismatches in the configuration and orientation of functional groups within the imprinted cavities (Figure 4). The rebinding process is sensitive to even minor changes, enhancing the precision of the sensor’s design. Such subtle distinctions illustrate that the sensor’s mechanism relies not only on the presence of common chemical moieties but also on the spatial arrangement of functional groups. ME (2,4,6-triamino-1,3,5-triazine) and SA are distinct chemical compounds with different molecular structures and properties. SA contains a hydroxyl group (−OH) and a carboxyl group (−COOH), while melamine has amino groups (−NH_2_) as part of its structure. In Figure 4, the sensor does not respond to ME. This lack of response further validates the selectivity of the sensor by underlining that the binding sites are highly specific to the molecular features of SA. This demonstrates that nonspecific adsorption, a crucial aspect of sensor reliability, is minimal.

**Figure 4:**
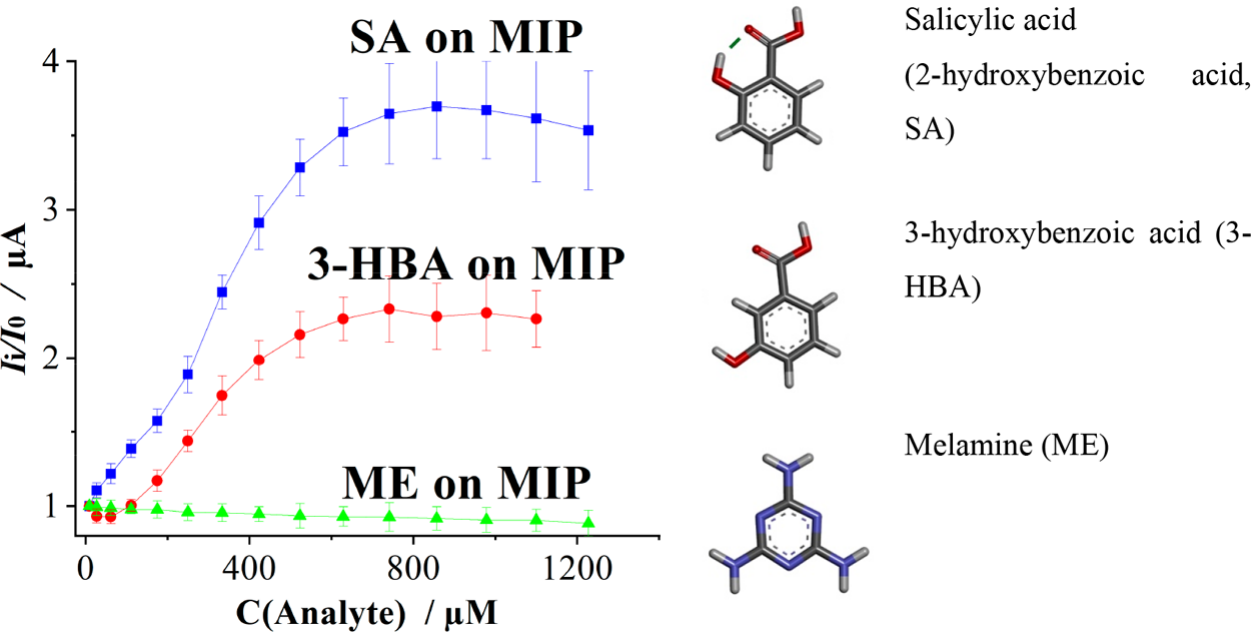
Evaluation of the developed electrochemical sensor’s selectivity for salicylic acid (SA) and structurally similar compounds, including 3-hydroxybenzoic acid and melamine, by using the DPV method. Molecular formulas were created with DFT/B3LYP/def2-TZVP.

Figure 4 demonstrates the MIP response to salicylic acid, 3-hydroxybenzoic acid and melamine. Table 1 summarizes electrochemical sensors based on MIP for salicylic acid, following criteria such as polymer type, template removal method, the list of interfering molecules, and LOD value. The electrochemical sensor proposed in Table 1 comprises various types of electrodes and polymers for forming the sensing layer. The present study demonstrates the overoxidation in an alkaline solution at a relatively low potential value. The current setup offers very low preparation costs, and with further adjustments, the LOD could be lowered to levels suitable for more sensitive detection applications. Despite this, the system proposed in this study has notable benefits, including a simplified sensor manufacturing process that can be reduced to just two steps: batch bulk polymerization and extraction, using small amounts of readily available materials.

**Table 1:** Summary of Electrochemical Sensors for Salicylic Acid Detection Based on Molecularly Imprinted Polymers^a^

detection technique	electrode and electrode modification	polymer and polymer modification	template removal method	sample	interfering molecules	LOD/M	refs
DPV	FTO/TiO_2_ NRAs	poly(MAA-*co*-EGDMA)	Methanol/acetic acid, V/V = 4:1 for 20 min gentle agitation	aspirin tablets		3.9 × 10^–8^ M	^20^
CV and DPV	GCE	Ppy	2-step template removal:	aspirin tablets	4-hydroxy-benzoic acid, 4-hydroxyl benzaldehyde, acetylsalicylic acid (aspirin).	3.5 × 10^–11^ M	^21^
			(1) fixed potential (+1.3 V) peroxide: electrolysis in 0.2 M Na_2_HPO_4_ for 10 min.				
			(2) alkali solution washing: in 0.2 M NaOH, ultrasonically for 3 min.				
SWV	GCE	PoPD	immersed in 0.1 M NaOH		4-hydroxybenzoic acid, 4-hydroxybenzaldehyde	2 × 10^–5^ M	^22^
CV and EIS	GCE/AuNP-Gr-Chi	poly(VP-*co*-EGDMA)	Methanol/acetic acid, V/V = 9:1 in a Soxhlet apparatus for 24 h.	wheat		1.3 × 10^–10^ M	^23^
DPV	Pt	Ppy	2-step template removal:		3-hydroxybenzoic acid, melamine	72 × 10^–6^ M	this study
			(1) electrochemical by potential cycling from 0 to +0.6 V vs Ag/AgCl_(3 M KCl)_, at a scan rate of 50 mV/s in a 0.1 M NaOH.				
			(2) immersing 0.1 M NaOH for 15 min.				

a AuNP—gold nanoparticle; Chi—chitosan; EGDMA—ethylene glycol dimethacrylate; EIS—electrochemical impedance spectroscopy; FTO—fluorine-doped tin oxide; GCE—glassy carbon electrode; Gr—graphene; MAA— methacrylic acid; PoPD— poly­(*o*-phenylenediamine); Pt— platinum electrode; SWV—square wave voltammetry; TiO_2_ NRAs—titanium oxide nanorod arrays; VP—4-vinylpyridine.

### 3.3 Results of DFT Calculations

#### 3.3.1 Hydrogen Bond analysis

After removing the imprinted salicylic acid from the MIP through extraction, an imprint is left that functions as a potential binding site for new molecules. This newly opened site could interact with different molecules, not just salicylic acid. To study this, we utilized DFT calculations at the B3LYP/def2-TZVP theory level to assess the selectivity of the binding site toward our small analyte, salicylic acid (maximum intramolecular distance in the optimized isolated structure of salicylic acid is 6.96 Å), and two potential interfering compounds: 3-hydroxybenzoic acid and melamine.

3-Hydroxybenzoic acid has a similar structure to salicylic acid and potentially could target the same binding sites. Meanwhile, melamine has a different molecular structure from salicylic acid and was chosen as a negative control. The binding sites created using salicylic acid should not be compatible with melamine. Zhihua et al.^21^ suggested that the interaction between salicylic acid and a pyrrole template functions through electrostatic attraction and hydrogen bonds. The DFT calculations provide insight into the hydrogen bonds within the system.

Figure 5 reveals that the binding site forms four conventional hydrogen bonds with salicylic acid, as well as one π-donor hydrogen bond and a carbon–hydrogen bond. The distances are depicted in Angstroms (Å). These interactions drive the MIP’s recognition ability toward the template, leading to selectivity and sensitivity. For 3-hydroxybenzoic acid, three conventional hydrogen bonds and a carbon–hydrogen bond are observed. The change in the hydroxyl group’s connectivity caused the loss of one hydrogen bond and the π-donor hydrogen bond. In the case of melamine, only one conventional hydrogen bond was detected. This explains how the tailor-made cavity binding site interacts with the tested molecules and is consistent with our electrochemical tests.

**Figure 5:**
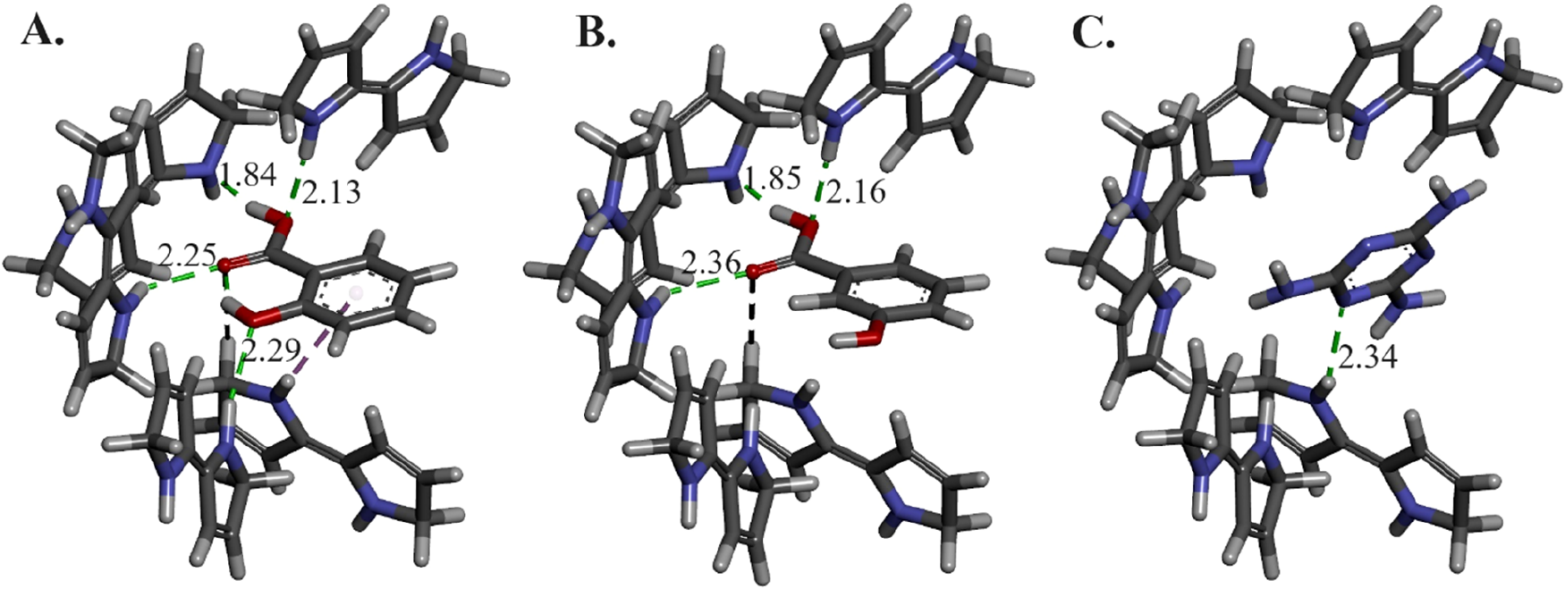
Intermolecular interactions of quinoid pyrrole dimers, modeled as the binding site, with (A) salicylic acid, (B) 3-hydroxybenzoic acid, and (C) melamine. The green dashed lines represent conventional hydrogen bonds and their corresponding distance in Å. The black dashed line represents carbon–hydrogen bonds, and the purple dashed line shows the π-donor hydrogen bond.

The obtained binding energies are −20.84, −18.39 and −11.80 kcal mol^–1^, for salicylic acid, 3-hydroxybenzoic acid, and melamine binding into the binding site, respectively. The binding energies demonstrate the greatest affinity of the tailor-made binding site toward the template and the least affinity for melamine.

Figure 4 has demonstrated that the electrochemical sensor responds to both salicylic acid and 3-hydroxybenzoic acid; however, the *I*_i_/*I*_0_ value for salicylic acid is higher. While the response shape for 3-hydroxybenzoic acid is similar, the detection threshold is significantly right-shifted by approximately 100 μM, resulting in a lower overall signal plateau. This can be explained by the weaker interactions binding site–analyte due to the loss of the hydrogen bond and the π-donor hydrogen bond.

The intramolecular hydrogen bond of salicylic acid remained steady and strong with 1.74 and 1.76 Å lengths in the isolated and complex states, respectively.

#### 3.3.2 Noncovalent Interaction Analysis

Further evaluation was done to analyze the details behind the MIP recognition ability. Therefore, noncovalent interaction analysis was performed to qualitatively compare the noncovalent interactions in the complexes. In this analysis, the function sign­(λ2)­ρ, which is the sign of the second largest eigenvalue of the electron density Hessian matrix (λ2) and electron density (ρ). The reduced density gradient (RDG) is obtained from the calculated electron density and its first derivative. RDG is utilized to demonstrate the deviation from a homogeneous electron distribution. Therefore, RDG has large positive values where the electron density exponentially approaches zero, far from the molecule.^44^ Along with sign­(λ2)­ρ, this function provides a good means for studying interactions.

The RDG scatter maps (Figure 6A–C) and graphs (Figure 6D–F) demonstrate the binding site complexed with salicylic acid (Figure 6A,6D), 3-hydroxybenzoic acid (Figure 6B,6E) and melamine (Figure 6C,6F). In the negative region of sign­(λ2)­ρ, where ρ is positive and λ2 is negative, spikes illustrate attractive hydrogen bond interactions that appear in the blue-green (weaker) and blue regions (stronger). There are more spikes in the binding site of the salicylic acid complex compared to the 3-hydroxybenzoic acid complex, indicating stronger intermolecular hydrogen bonding and intramolecular hydrogen bonding in salicylic acid. Meanwhile, no spikes in the blue region of the melamine indicate no strong hydrogen bond formation (Figure 6C).

**Figure 6:**
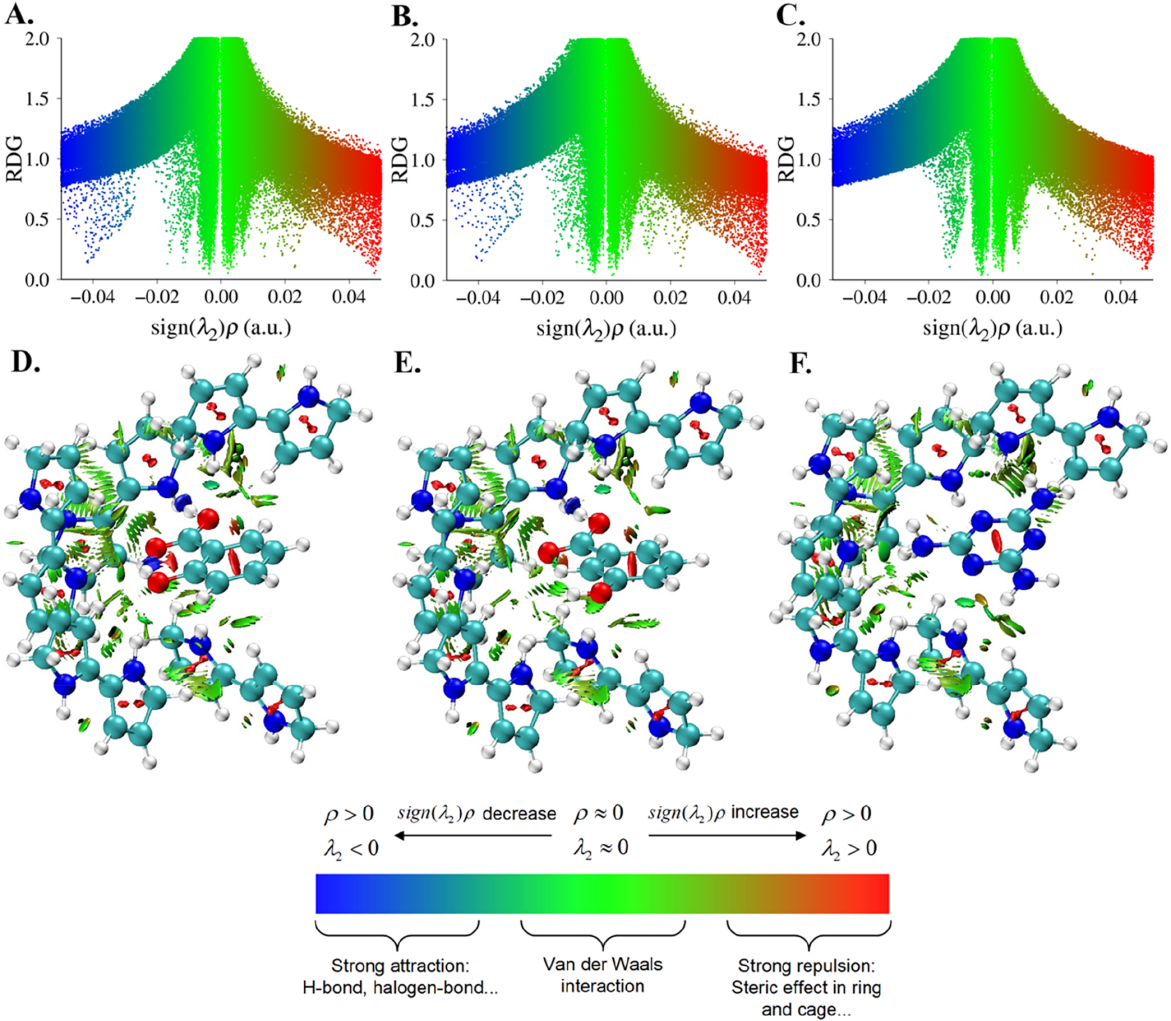
RDG scatter maps (A–C) and RDG graphs (D–F, isosurface 0.5) of complexes comprising the binding site with (A) salicylic acid, (B) 3-hydroxybenzoic acid, and (C) melamine. Legend: Blue and blue-green (negative) sign­(λ2)­ρ region shows attractive hydrogen bonds. The green (middle, near zero) part represents the Van Der Waals force, and the red (positive) part is the repulsive force.

This result confirms our previous evaluation of hydrogen bond formation in the complexes and their distances as a measure of their strength, as well as the correlation between the signal strength observed in our electrochemical sensor testing. The spikes in the green region, where λ2 and ρ are near zero, indicate van Der Waals interactions, including dipole–dipole, dipole–induced dipole, and London dispersion interactions, which are widespread in all complexes. This dominance is evident in Figure 6D–F.

However, the spikes represent repulsive forces, such as steric effects in the red regions, where λ2 and ρ are positive. Despite the similarity of all complexes in posing steric effects at the center of the rings, more spikes are observable for the binding site complexed with salicylic acid and 3-hydroxybenzoic acid. Figure 6D,6E show that these spikes stemmed from intramolecular steric hindrance between their functional groups and the rings, respectively. However, melamine did not have the repulsive force between its amino groups due to their spatial arrangement, confirming its relative inertness in the system.

The similarity between scatter plots and graphs of salicylic acid and 3-hydroxybenzoic acid isomers interacting with the binding site suggests a recognition capability for aromatic carboxylic acid derivatives. Any sensor manufactured using this platform will exhibit some degree of parasitic sensor interference and false positive responses in the presence of nontarget aromatic carboxylic acids in the system. However, based on our electrochemical testing, this interaction is expected to be negligible at lower concentrations.

That is noteworthy, but the intramolecular hydrogen bond in salicylic acid did not interrupt the imprinting process. Hydrogen bonds and van der Waals interactions play a significant role in the host–guest functional molecular systems of the MIP-template complex. Numerous optimized donor–acceptor interactions, which were complementary to the conformation of salicylic acid as a template, provided the specific recognition of the template. Therefore, the calculation demonstrated the sensor’s capability to recognize salicylic acid selectively.

## 4 Conclusions

This study demonstrates the development of an electrochemical molecularly imprinted polymer (MIP)-based sensor utilizing conducting polymer polypyrrole (Ppy) for the selective detection of salicylic acid. This research highlights the use of Ppy due to its electrical conductivity and stability and underscores the sensor’s selectivity and sensitivity toward salicylic acid. The fabrication process involved imprinting salicylic acid into a Ppy matrix and creating specific binding sites upon removal of the template. Selectivity analysis confirmed the sensor’s superior affinity for salicylic acid over other compounds, such as 3-hydroxybenzoic acid and melamine. A deeper investigation into the binding interactions using electrostatic potential maps (EPM) revealed a complex network of hydrogen bonds, π–π stacking, and π-donor interactions within the binding site. These interactions were crucial in forming a stable and specific recognition environment for salicylic acid. The MIP sensor’s performance was validated with a limit of detection (LOD) of 72 μM and a limit of quantification (LOQ) of 217 μM. Therefore, this study provides insights into the development of electrochemical salicylic acid sensors based on MIP. Further optimizations could enhance its performance for applications requiring accurate and selective detection of this analyte.

## Supplementary Material



## Data Availability

Data demonstrated in this study will be available from the corresponding author upon reasonable request.

## Notes

Cyclic voltammograms of the Pt electrode in a Britton-Robinson buffer solution (pH = 7) with 0.05 M SA registered by potential cycling, within the range of −0.4 to +1 V vs Ag/AgCl_(3 M KCl)_ at the scan rate of 50 mV/s (Figure S1) (PDF)
