# Supplementary material for: Electrochemical Salicylic Acid Sensor Based on Molecularly Imprinted Polypyrrole
Source: ACS Appl Mater Interfaces. 2025 Oct 2;17(41):57475–85. doi: 10.1021/acsami.5c11951 (PMC12532085; doi:10.1021/acsami.5c11951)
Supplement: Supplementary file 1 [file am5c11951_si_001.pdf]

Supporting information

Electrochemical salicylic acid sensor based on molecularly imprinted polypyrrole

Greta Zvirzdine [1], Sarunas Zukauskas [1], Alma Rucinskiene [2], Enayat Mohsenzadeh [1], Raimonda Boguzaite [1], Almira Ramanaviciene [3], Maksym Pogorielov [5,6], Vilma Ratautaite [1], Arunas Ramanavicius [1,4,] *

[1] Department of Nanotechnology, State Research Institute Center for Physical Sciences and Technology (FTMC), Sauletekio Ave. 3, LT-10257, Vilnius, Lithuania;

[2] Department of Electrochemical Material Science, State Research Institute Center for Physical Sciences and Technology (FTMC), Sauletekio Ave. 3, LT-10257, Vilnius, Lithuania;

[3] NanoTechnas – Center of Nanotechnology and Material Science, Institute of Chemistry, Faculty of Chemistry and Geosciences, Vilnius University (VU), Naugarduko Str. 24, LT-03225, Vilnius, Lithuania;

[4] Department of Physical Chemistry, Institute of Chemistry, Faculty of Chemistry and Geosciences, Vilnius University (VU), Naugarduko Str. 24, LT-03225, Vilnius, Lithuania.

[5] Biomedical Research Centre, Sumy State University, Kharkivska street 116, 40007 Sumy, Ukraine;

[6] Institute of Atomic Physics and Spectroscopy, University of Latvia, Jelgavas iela 3, LV-1004 Riga, Latvia.

Corresponding author: arunas.ramanavicius@chf.vu.lt (A. Ramanavicius, VU).

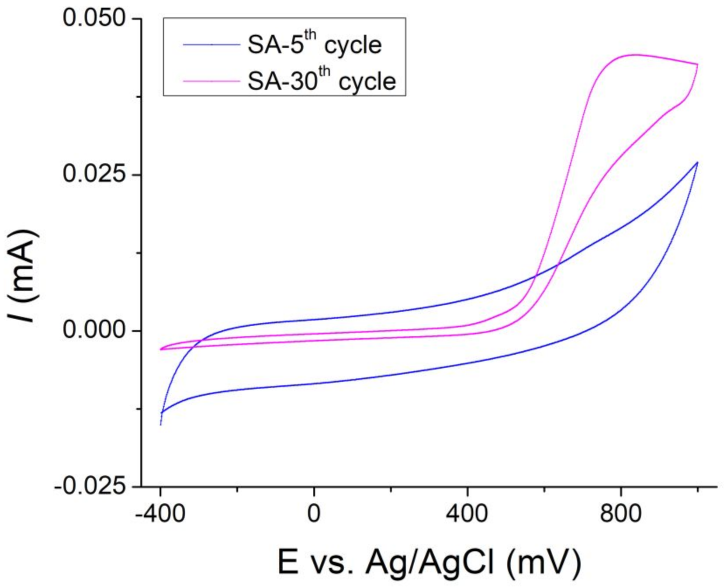

Figure S1. Cyclic voltammograms of the Pt electrode in a Britton-Robinson buffer solution (pH=7) with 0.05 M SA registered by potential cycling, within the range of –0.4 to +1 V vs. Ag/AgCl$_{(3M\ KCl)}$ at the scan rate of 50 mV/s.
